# Association between blood lipids and diabetes mellitus in older Chinese adults aged 65 years or older: a cross-sectional analysis of residents’ electronic health records

**DOI:** 10.1186/s12944-024-02160-7

**Published:** 2024-06-04

**Authors:** Tianxiang Lin, Yanrong Zhao, Qing Yang, Wei Wang, Xuewen Jiang, Yinwei Qiu

**Affiliations:** Zhejiang Provincial Center for Disease Control and Prevention, Dept. of Public Health Surveillance & Advisory, No.3399. Binsheng Road, Binjiang district, Hangzhou, 310051 PR China

**Keywords:** Diabetes, Blood lipids, Restricted cubic splines, Cross-sectional analysis

## Abstract

**Aim:**

This study aimed to investigate how blood lipids are associated with diabetes among older Chinese adults.

**Methods:**

3,268,928 older Chinese adults without known diabetes were included. Logistic regression and restricted cubic spline (RCS) models were conducted to study associations between blood lipids (total cholesterol [TC], triglycerides [TG], low-density lipoprotein cholesterol [LDL-C], and high-density lipoprotein cholesterol [HDL-C]) and diabetes.

**Results:**

202,832 diabetes cases were included. Compared with the lowest quintiles, TC, TG, and LDL-C in the highest quintiles showed a higher diabetes prevalence risk and HDL-C presented a lower risk in multivariate-adjusted logistic regression models. Odds ratios (ORs) and 95% confidence intervals (95% CIs) for the highest quintiles of TC, TG, and HDL-C were 1.39 (1.37–1.41), 2.56 (2.52–2.60), and 0.73 (0.72–0.74), respectively. For LDL-C, 3–5% lower risk was found in the second and third quintiles, and 4–23% higher risk was found in the fourth and fifth quintiles. RCS curves showed a non-linear relationship between each blood lipid parameters and diabetes (*P*-non-linear < 0.001). TG and HDL-C curves presented monotonically increasing and L-shaped patterns, respectively, whereas TC and LDL-C curves exhibited a J-shaped pattern. When TC < 4.04 mmol/L or LDL-C < 2.33 mmol/L, ORs of diabetes increased with the decrease of corresponding indexes. However, after excluding participants with lower LDL-C, the J-shaped association with TC disappeared.

**Conclusions:**

This study demonstrates non-linear associations between lipids and diabetes. Low cholesterol levels are associated with a high risk of diabetes. The cholesterol paradox should be considered during lipid-lowering treatments.

**Supplementary Information:**

The online version contains supplementary material available at 10.1186/s12944-024-02160-7.

## Introduction

Diabetes mellitus is one kind of metabolic disease characterized by hyperglycemia. It is a leading cause of death and shortened life expectancy and seriously affects the health of people worldwide. In 2017, the global prevalence of diabetes stood at 476.0 million, resulting in 1.37 million deaths and an estimated disability-adjusted life-years related to the disease was 67.9 million [1]. It is estimated that the number of people with diabetes will rise to approximately 642 million by 2040 [2].

Blood lipids is a collective term for cholesterol, triglycerides (TG), and lipids (such as phospholipids) in the serum, and the common test items for blood lipids include total cholesterol (TC), TG, low-density lipoprotein cholesterol (LDL-C), and high-density lipoprotein cholesterol (HDL-C). The role of dyslipidemia, an established risk factor for diabetes, in the prevalence and control of diabetes has been previously described [3, 4]. Additionally, lowering non-HDL-C levels, such as TG and LDL-C, could be used as a primary or secondary prevention strategy for cardiovascular disease [5–7]. The “lower is better” strategy has gained popularity in blood lipid management. Cholesterol is an essential nutrient for maintaining normal human physiological functions, such as cell membrane structure [8], cell signaling [9], and hormone production [10]. Complex mechanisms maintain blood lipid levels within a physiological range, and the dysregulation of these mechanisms can lead to elevated or decreased tissue cholesterol levels, resulting in embryonic or adult diseases [11]. Additionally, a number of real-world studies [12] and randomized controlled trials [13] have demonstrated a paradoxical link between diabetes and blood lipid levels. A meta-analysis of 13 trials related to statin demonstrated the promoting effect of statin treatment on the risk of diabetes and found that the incidence risk of diabetes expanded by 9% in the treatment group [14]. In another cross-sectional study, a correlation between significantly lower TC and LDL levels and a higher prevalence of diabetes was observed in older people, regardless of the use of lipid-lowering medications [15].

Although the cholesterol paradox has attracted increasing attention, the association between blood lipid levels and diabetes in older Chinese adults remains unclear, even though older adults are prone to dyslipidemia and diabetes [16, 17]. Moreover, previous studies only focused on one or two blood lipid indicators [18] or divided blood lipid status into two groups (normal or dyslipidemia) [3]. No systematic and detailed analyses have been conducted on traditional blood lipid parameters, particularly the dose–response relationships between blood lipids and diabetes and the contribution of different lipid components to the cholesterol paradox.

Therefore, this study aimed to explore the potential associations of blood lipids with diabetes among older Chinese adults and to provide a more comprehensive understanding of TC, TG, LDL-C, and HDL-C in association with diabetes.

## Methods

### Study population

China’s Basic Public Health Services Project is a public welfare project for primary healthcare aimed at all Chinese people, among which physical examination of the elderly is an important service. At the same time as providing free health services to the Chinese people through China’s Basic Public Health Services Project, a huge database called Residents’ Electronic Health Records has been established [19].

In total, 5,697,488 residents aged 65 years or older attending an annual physical examination in Zhejiang Province, China, in 2022 were initially included in this study. Participants who missed one or more research variables and/or had outliers (exceeding three times the standard deviation, clearly not meeting clinical standards) were excluded (*n* = 1,331,487). In addition, participants who had already been diagnosed with diabetes were excluded from the study (*n* = 1,097,073) to avoid the influence of drug use and behavioral interaction on levels of fasting blood glucose, blood lipids, and other variables. Ultimately, 3,268,928 participants with complete and logical parameters and without known diabetes were included.

### Data collection

Demographic, clinical, and behavioral data were extracted from the Residents’ Electronic Health Records, 2022. The demographic data included age, sex, educational attainment, and marital status. The clinical data included body height, weight, waist circumference (WC), fasting blood glucose (FBG), systolic blood pressure (SBP), diastolic blood pressure (DBP), alanine aminotransferase (ALT), aspartate aminotransferase (AST), total bilirubin (TBil), serum creatinine (Scr), blood urea nitrogen (BUN), TC, TG, LDL-C, and HDL-C. Behavioral data included smoking status, alcohol consumption status, and physical exercise.

### Variables definition

Referring to the Chinese guidelines for type 2 diabetes, FBG ≥ 7.0 mmol/L was defined as diabetes in this study [20].

Body mass index (BMI) was calculated as body weight (kg) divided by body height (m) squared. BMI < 18.5 kg/m^2^, ≥ 24 kg/m^2^, and ≥ 28 kg/m^2^ was defined as low BMI, overweight and obesity, respectively [21]. WC ≥ 85 cm for men and ≥ 80 cm for women were defined as central obesity [21].

Behavioral variables were defined based on actual information provided by residents in the Residents’ Electronic Health Records, 2022. The smoking status was divided into three categories: never (participants who reported not smoking), regular smokers (participants who smoke at least one cigarette per day), and former smokers (participants who stopped smoking). Alcohol consumption was divided into three categories: never (participants who reported not drinking), drinkers (participants who reported drinking occasionally or regularly), and former drinkers (participants who stopped drinking). Physical exercise was defined as conscious engagement in at least 30 min of physical activity outside of work for > 3 days a week and was divided into two categories in this study: no and yes.

### Statistical analysis

To analyze the association between blood lipids and diabetes, logistic regression models were used. TC, TG, LDL-C, and HDL-C levels were categorized into five groups based on their respective distributions. In the multivariate models, potential confounders were adjusted for age, sex, educational attainment, marital status, BMI, central obesity, SBP, DBP, ALT, AST, TBil, Scr, BUN, smoking status, physical exercise, and alcohol consumption. In addition, the TC, TG, LDL-C, and HDL-C were highly correlated. Therefore, to avoid multicollinearity in the statistical analysis, TC, TG, LDL-C and HDL-C were entered into the statistical models solely.

Restricted cubic splines (RCS) were used to flexibly model the associations of TC, TG, LDL-C, and HDL-C with diabetes. In spline models, four knots located at the 5th, 35th, 65th, and 95th percentiles were set up, and the comparison between the log-likelihood of a model with spline variables and the log-likelihood of a model with only a linear effect of the covariate was performed to test potential nonlinearity [22]. As the associations of TC, LDL-C, and HDL-C were approximately log-linear below and above their inflection points, the odds ratios (ORs) per 1 unit increase in TC, LDL-C, and HDL-C were calculated through logistic regression models. Given the similar shape between the TC-diabetes and LDL-C-diabetes relationships in the pre-phase analysis and the biological inclusion of LDL-C in TC, we examined how the shape of the TC-diabetes association changes after excluding data with low LDL-C (defined as those below the 2.5th, 5th, and 10th percentiles of total data).

Subgroup analysis was performed to study the associations between blood lipids and diabetes in various subgroups stratified by sex (male and female) and age (65-, 70-, 75- and 80-). In the subgroup analysis, multivariate logistic regression models were used, and confounding factors were fully adjusted, except for grouping factors. For sensitivity analysis, participants with glycosylated hemoglobin (HbA1c) data were extracted, and HbA1c ≥ 6.5% was defined as diabetes [23]. In total, 1,054,679 participants, including 67,949 patients with diabetes (HbA1c ≥ 6.5%), were included in the RCS models, with fully adjusted confounding factors to assess the relationship between blood lipids and diabetes.

All analyses were performed using R 4.3.0 for Windows 64-bit (R Foundation for Statistical Computing, Vienna, Austria. https://www.R-project.org/), and the significance level (alpha) was set at 0.05. RCS analysis for graphical displays was performed using the ‘rcssci’ package (Zhiqiang Nie (2023), R package version 0.4.0, https://cran.r-project.org/web/packages/rcssci/index.html).

## Results

### Study participants

In total, 3,268,928 older adults (≥ 65) were included in the analysis. The characteristics of participants were presented in Table 1, according to FBG levels (Normal [< 7 mmol/L] and Diabetes [≥ 7 mmol/L]). The average age of the participants was 72.60 years, with a standard deviation (SD) of 6.10 years. Additionally, 46.44% of the participants were male. The mean concentrations of TC, TG, LDL-C, and HDL-C in total participants population were 4.97 ± 1.06 mmol/L, 1.58 ± 1.00 mmol/L, 2.85 ± 0.87 mmol/L, and 1.44 ± 0.41 mmol/L, respectively, with corresponding SDs. FBG concentrations of 202,832 (6.20%) participants were ≥ 7 mmol/L, and compared with the normal FBG group, the levels of TC, TG, LDL-C were higher, and the levels of HDL-C were lower in the diabetes group (*P* < 0.001).

**Table 1:** Participant characteristics according to fasting blood glucose (electronic health records of residents aged 65 or older, 2022)

Variables	All participants *N* = 3,268,928^1^	Normal *N* = 3,066,096 (93.80%)^1^	Diabetes *N* = 202,832 (6.20%)^1^	*P*-value^2^
**Sex, n (%)**				< 0.001
Male	1,518,008(46.44%)	1,420,614(46.33%)	97,394(48.02%)	
Female	1,750,920(53.56%)	1,645,482(53.67%)	105,438(51.98%)	
**Age, n (%)**				< 0.001
65-	1,239,677(37.92%)	1,165,745(38.02%)	73,932(36.45%)	
70-	989,806(30.28%)	929,543(30.32%)	60,263(29.71%)	
75-	581,752(17.80%)	544,820(17.77%)	36,932(18.21%)	
80-	457,693(14.00%)	425,988(13.89%)	31,705(15.63%)	
**Educational attainment, n(%)**				< 0.001
Illiterate and primary	2,185,840(66.87%)	2,057,436(67.10%)	128,404(63.31%)	
Junior and senior	604,521(18.49%)	566,305(18.47%)	38,216(18.84%)	
College degree or above	51,418(1.57%)	48,248(1.57%)	3,170(1.56%)	
Unknown	427,149(13.07%)	394,107(12.85%)	33,042(16.29%)	
**Marital status, n(%)**				< 0.001
Single	365,485(11.18%)	341,204(11.13%)	24,281(11.97%)	
Married	2,524,380(77.22%)	2,376,295(77.50%)	148,085(73.01%)	
Unknown	379,063(11.60%)	348,597(11.37%)	30,466(15.02%)	
**SBP (mm Hg)**	138.44 ± 18.58	138.23 ± 18.54	141.70 ± 18.85	< 0.001
**DBP (mm Hg)**	78.80 ± 10.40	78.75 ± 10.39	79.57 ± 10.57	< 0.001
**BMI**				< 0.001
normal	1,721,638(52.67%)	1,637,475(53.41%)	84,163(41.49%)	
Low BMI	171,591(5.25%)	165,847(5.41%)	5,744(2.83%)	
Overweight	1,094,285(33.48%)	1,010,901(32.97%)	83,384(41.11%)	
Obesity	281,414(8.61%)	251,873(8.21%)	29,541(14.56%)	
**Central obesity, n (%)**	1,091,009(33.38%)	996,878(32.51%)	94,131(46.41%)	< 0.001
**ALT (U/L)**	20.8 ± 15.27	20.48 ± 14.79	25.59 ± 20.62	< 0.001
**AST (U/L)**	25.31 ± 15.18	25.20 ± 14.74	27.08 ± 20.70	< 0.001
**TBil (µmol/L)**	14.21 ± 6.5	14.18 ± 6.46	14.68 ± 7.07	< 0.001
**Scr (µmol/L)**	72.89 ± 26.3	72.86 ± 26.11	73.32 ± 29.07	< 0.001
**BUN (mmol/L)**	5.98 ± 2.84	5.98 ± 2.83	6.04 ± 2.98	< 0.001
**TC (mmol/L)**	4.97 ± 1.06	4.96 ± 1.06	5.11 ± 1.14	< 0.001
**TG (mmol/L)**	1.58 ± 1.00	1.56 ± 0.97	1.96 ± 1.32	< 0.001
**LDL_C (mmol/L)**	2.85 ± 0.87	2.84 ± 0.86	2.94 ± 0.92	< 0.001
**HDL_C (mmol/L)**	1.44 ± 0.41	1.45 ± 0.41	1.38 ± 0.40	< 0.001
**Physical exercise, n (%)**	1,065,837(32.61%)	996,407(32.50%)	69,430(34.23%)	< 0.001
**Smoking status**				< 0.001
Never	2,595,250(79.39%)	2,432,645(79.34%)	162,605(80.17%)	
Regular smoker	522,539(15.99%)	491,533(16.03%)	31,006(15.29%)	
Former smoker	151,139(4.62%)	141,918(4.63%)	9,221(4.55%)	
**Alcohol consumption status**				< 0.001
Never	2,490,705(76.19%)	2,338,751(76.28%)	151,954(74.92%)	
Drinker	729,752(22.32%)	681,598(22.23%)	48,154(23.74%)	
Former drinker	48,471(1.48%)	45,747(1.49%)	2,724(1.34%)	

1 Data are presented as n (%) or mean ± SD. Binary variables, central obesity and physical exercise, are listed the data of “Yes” only.

2 Differences of categorical variables between groups were examined with Pearson’s Chi-squared test, while numerical variables were examined with t-test.

### Logistic regression modeling to assess associations between blood lipid indicators and diabetes

Table 2 presents the associations between blood lipid parameters and diabetes. Multivariate-adjusted logistic regression models showed a positive relationship between TG and diabetes, whereas diabetes was negatively correlated with HDL-C. Compared with the lowest quintile of TG, the highest quintile had an OR of 2.56 for diabetes, with a 95% confidence interval (95% CI) of 2.52–2.60. For HDL-C, compared with the lowest quintile, an OR of 0.72 (0.71–0.73) was obtained among participants in the highest quintile. Notably, LDL-C showed a J-shaped association with diabetes in the multivariate-adjusted logistic regression models. There was a 3–5% lower prevalence risk of diabetes among older adults in the second and third quintiles, whereas those in the fourth and fifth quintiles had a 4–23% higher prevalence risk of diabetes than those in the lowest quintile of LDL-C. As for TC, when compared to those in the first quintile, the risk of diabetes prevalence did not exhibit a significant increase among participants in the second quintile. However, for those in the third to fifth quintiles, the risk of diabetes prevalence increased by 4 to 38%.

**Table 2:** OR (95% CI) of diabetes in relation to TC, TG, LDL-C and HDL-C (electronic health records of residents aged 65 or older, 2022)

				Model1			Model2			Model3	
	Normal (n)	Case (n)	Prevalence (%)	OR (95% CI)	*P*-value		OR (95% CI)	*P*-value		OR (95% CI)	*P*-value
TC											
1 (lowest)	615,529	37,190	5.70	1 (reference)			1 (reference)			1 (reference)	
2	610,837	35,328	5.47	0.98 (0.97-1.00)	0.04		0.99 (0.97-1.00)	0.156		0.99 (0.97-1.00)	0.116
3	614,512	37,400	5.74	1.04 (1.03–1.06)	< 0.001		1.04 (1.02–1.06)	< 0.001		1.04 (1.02–1.05)	< 0.001
4	622,109	41,920	6.31	1.16 (1.14–1.18)	< 0.001		1.13 (1.12–1.15)	< 0.001		1.13 (1.11–1.15)	< 0.001
5 (highest)	603,109	50,994	7.80	1.46 (1.44–1.48)	< 0.001		1.39 (1.37–1.41)	< 0.001		1.38 (1.36–1.40)	< 0.001
TG											
1 (lowest)	593,104	22,463	3.65	1 (reference)			1 (reference)			1 (reference)	
2	656,897	31,083	4.52	1.30 (1.28–1.33)	< 0.001		1.22 (1.20–1.24)	< 0.001		1.22 (1.20–1.24)	< 0.001
3	568,323	33,705	5.60	1.65 (1.62–1.68)	< 0.001		1.46 (1.43–1.48)	< 0.001		1.46 (1.43–1.49)	< 0.001
4	658,006	48,773	6.90	2.09 (2.05–2.12)	< 0.001		1.76 (1.73–1.79)	< 0.001		1.76 (1.73–1.79)	< 0.001
5 (highest)	589,766	66,808	10.18	3.22 (3.17–3.27)	< 0.001		2.56 (2.52–2.60)	< 0.001		2.56 (2.52–2.60)	< 0.001
LDL-C											
1 (lowest)	606,292	37,941	5.89	1 (reference)			1 (reference)			1 (reference)	
2	622,287	36,329	5.52	0.93 (0.91–0.94)	< 0.001		0.95 (0.93–0.96)	< 0.001		0.95 (0.93–0.96)	< 0.001
3	618,841	37,381	5.70	0.98 (0.96–0.99)	0.003		0.97 (0.96–0.98)	< 0.001		0.97 (0.96–0.98)	< 0.001
4	607,079	40,592	6.27	1.08 (1.07–1.10)	< 0.001		1.04 (1.02–1.05)	< 0.001		1.04 (1.02–1.05)	< 0.001
5 (highest)	611,597	50,589	7.64	1.33 (1.32–1.35)	< 0.001		1.23 (1.21–1.25)	< 0.001		1.23 (1.21–1.25)	< 0.001
HDL-C											
1 (lowest)	568,534	47,316	7.68	1 (reference)			1 (reference)			1 (reference)	
2	635,554	47,909	7.01	0.92 (0.91–0.94)	< 0.001		0.93 (0.92–0.94)	< 0.001		0.93 (0.91–0.94)	< 0.001
3	598,239	39,357	6.17	0.81 (0.80–0.82)	< 0.001		0.85 (0.84–0.86)	< 0.001		0.84 (0.83–0.85)	< 0.001
4	629,901	36,033	5.41	0.70 (0.69–0.71)	< 0.001		0.77 (0.76–0.79)	< 0.001		0.77 (0.76–0.78)	< 0.001
5 (highest)	633,868	32,217	4.84	0.62 (0.61–0.63)	< 0.001		0.73 (0.72–0.74)	< 0.001		0.72 (0.71–0.73)	< 0.001

TC, TG, LDL-C and HDL-C were categorized into five groups based on their respective distributions.

Model1: adjusted for age (65-, 70-, 75, 80-), sex (male, female), educational attainment (illiterate and primary, junior and senior, college degree or above, unknown) and marital status (single, married, unknown).

Model2: model1 adjusted for BMI (normal, low BMI, overweight, obesity), central obesity (no, yes), SBP, DBP, ALT, AST, TBil, Scr and BUN.

Model3: model2 adjusted for smoking status (never, regular smoker, former smoker), physical exercise (no, yes) and alcohol consumption status (never, drinker, former drinker).

### RCS to assess associations between blood lipid parameters and diabetes

**Fig. 1:**
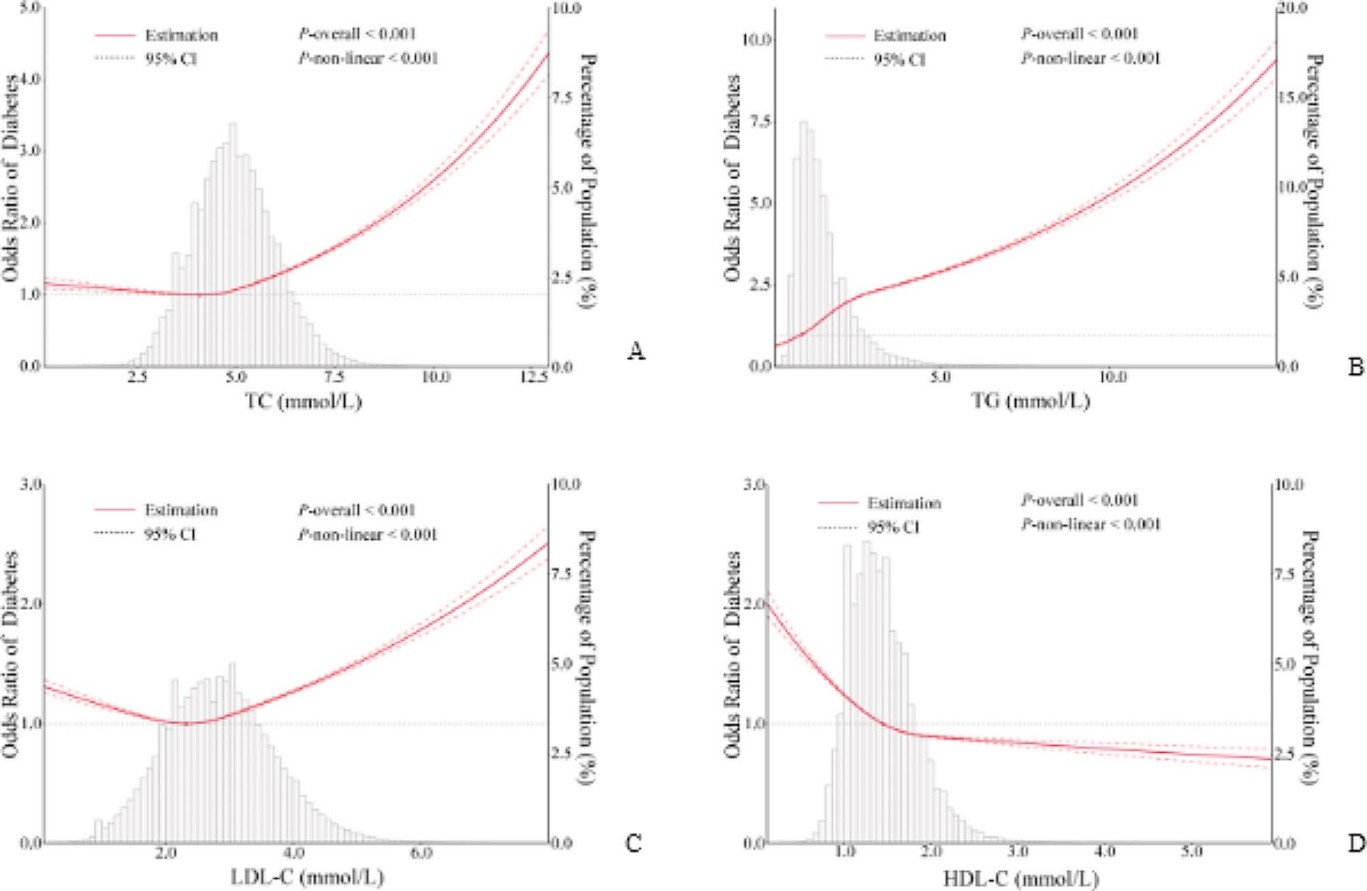
Association of TC, TG, LDL-C and HDL-C with diabetes. A: TC and diabetes; B: TG and diabetes; C: LDL-C and diabetes; D: HDL-C and diabetes. Odds ratios are indicated by red solid lines and border of 95% CIs by red dashed lines (left coordinate axis). Histograms represent the percentage of each group to the total population (right coordinate axis). Reference point is 20th centile of TG, inflection point of HDL-C and lowest value for each of TC and LDL-C, with knots placed at 5th, 35th, 65th, and 95th centiles of each TC, TG, LDL-C and HDL-C distribution. All models were adjusted for confounders in Table 2

RCS was used to model and visualize the associations of TC, TG, LDL-C, and HDL-C with diabetes in older adults, and these four blood lipid indicators all exhibited non-linear relationships with diabetes (*P*-non-linear < 0.001, Fig. 1). A positive relationship was observed between TG and the OR of diabetes (Fig. 1B), whereas an L-shaped relationship between HDL-C and diabetes was presented in the RCS (Fig. 1D). The OR of diabetes decreased rapidly within the lower range of HDL-C, and when the HDL-C was above 1.44 mmol/L, the reduction trend became relatively flat. Below 1.44 mmol/L, the OR per 1 unit higher HDL-C was 0.61 (0.59–0.63), and it was 0.90 (0.89–0.92) above 1.44 mmol/L (Fig. 1D; Table 3).

**Fig. 2:**
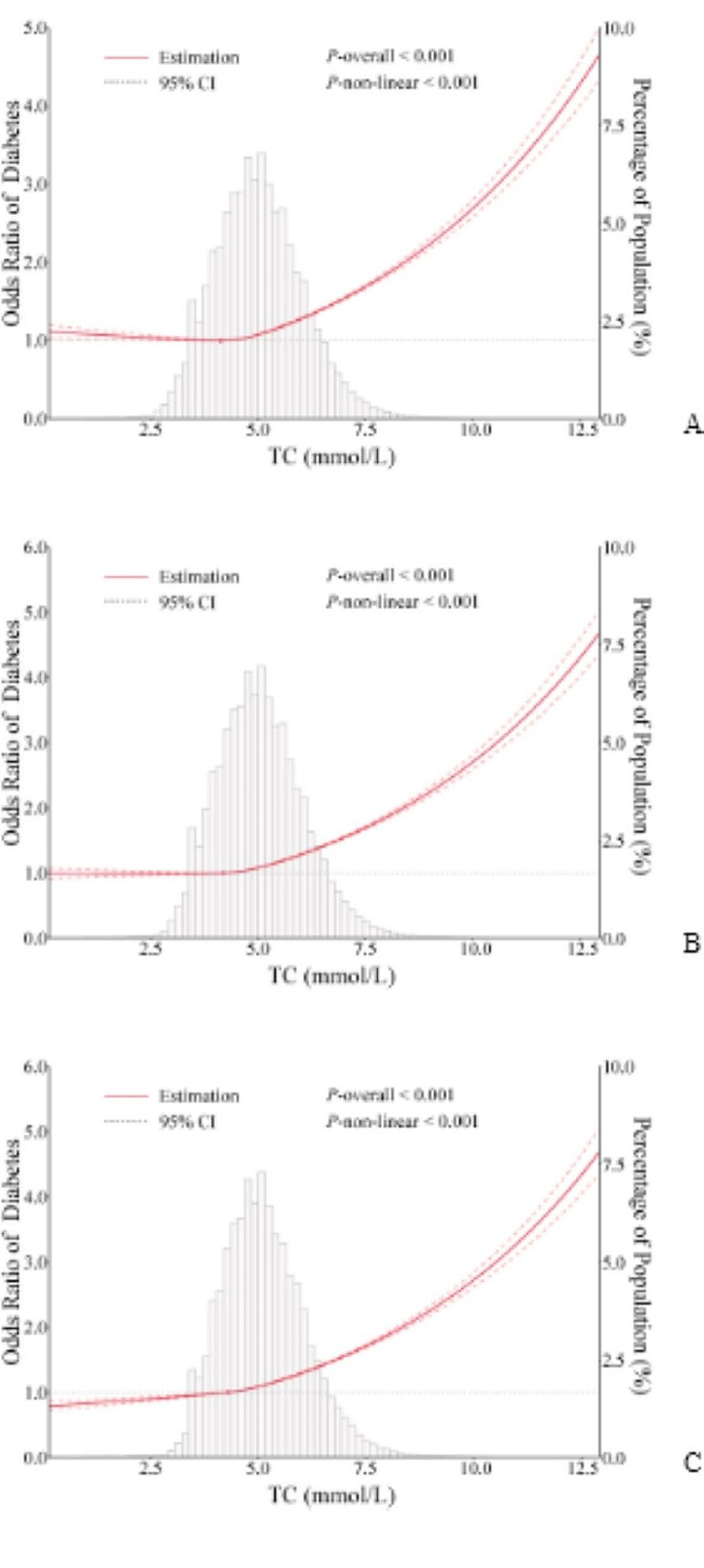
ORs (95% CI) of diabetes according to TC (exclusion of low LDL-C). A: excluded participants with LDL-C below 2.5th centile; B: excluded participants with LDL-C below 5th centile; C: excluded participants with LDL-C below 10th centile. Odds ratios are indicated by red solid lines and border of 95% CIs by red dashed lines (left coordinate axis). Histograms represent the percentage of each group to the total population (right coordinate axis). Reference point is 20th centile of each TC after exclusion of low LDL-C, with knots placed at 5th, 35th, 65th, and 95th centiles. All models were adjusted for confounders in Table 2

Strong J-shaped associations for TC and LDL-C between diabetes were observed using the RCS (Fig. 1A and C). A decrease in OR was observed until 4.04 mmol/L of TC, and the OR was 0.97 (0.95–1.00) per 1 mmol/L increase of TC; when TC was above 4.04 mmol/L, the OR was 1.17 (1.17–1.18) per 1 mmol/L increase of TC (Fig. 1A; Table 3). The plot demonstrated a considerable decrease in the OR within the lower range of LDL-C levels, reaching its nadir at approximately 2.33 mmol/L, before gradually increasing thereafter. Below 2.33 mmol/L, the OR for every 1 mmol/L increase of LDL-C was 0.88 (0.86–0.90), and it was 1.18 (1.17–1.18) after above 2.33 mmol/L of LDL-C (Fig. 1C; Table 3). The relationship between TC and diabetes was examined after excluding the participants with low LDL-C levels. When participants below the 2.5th percentile of LDL-C were excluded, the J-shaped relationship between TC and diabetes weakened. After excluding participants with low LDL-C concentrations (< 5th and 10th), the J-shaped association between TC and diabetes disappeared and transformed into a positive correlation (Fig. 2). However, a slight change was observed in the J-shaped association as participants with low HDL-C levels were gradually excluded; they did not disappear (Supplementary Figure A).

### Subgroup and sensitivity analysis

The J-shaped association between TC and diabetes was stronger in females than in males and stronger in the 65- than in higher age groups (70-, 75-, and 80-); similar associations for TG, LDL-C, and HDL-C were found across all subgroups in the subgroup analysis (Supplementary Tables A and B). The findings of this study remained robust in the sensitivity analysis. After adjusting for the confounding factors presented in Table 2, the RCS curves of these four traditional blood lipid parameters were similar to those shown in Fig. 1 (Supplementary Figure B).

**Table 3:** OR (95% CI) of diabetes for every 1 mmol/L increase of TC, LDL-C and HDL-C, stratified by the inflection point of HDL-C and lowest value for each of TC and LDL-C according to RCS (electronic health records of residents aged 65 or older, 2022)

	OR	95% CI	*P*-value
TC (mmol/L)			
< 4.04	0.97	0.95, 1.00	0.035
≥ 4.04	1.17	1.17, 1.18	< 0.001
LDL-C (mmol/L)			
< 2.33	0.88	0.86, 0.90	< 0.001
≥ 2.33	1.18	1.17, 1.18	< 0.001
HDL-C (mmol/L)			
< 1.44	0.61	0.59, 0.63	< 0.001
≥ 1.44	0.90	0.89, 0.92	< 0.001

According to RCS, when TC and LDL-C are 4.04 and 2.33 mmol/L respectively, their respective ORs are the lowest; the inflection point of HDL-C is at 1.44 mmol/L. Adjusted for confounding factors shown in Table 2.

## Discussion

In this cross-sectional study of 3,268,928 older Chinese adults, the shapes of the associations between common blood lipid parameters (TC, TG, LDL-C, and HDL-C) and diabetes were determined. The relationship between the four blood lipid indicators and diabetes was non-linear with different shapes. A strong positive association was observed between TG levels and diabetes. In contrast, a negative relationship was observed between HDL-C levels and diabetes, and the OR curve was L-shaped. Both TC and LDL-C levels showed J-shaped associations with diabetes, with an inverse correlation before the inflection point and a positive correlation after the inflection point.

Many epidemiological studies have explored the association between blood lipid parameters and diabetes, and Low HDL-C and high TG have been proven to be established risk factors for diabetes in several previous studies [4, 18, 24, 25]. An observational study of 5,012 participants, which was based on two cross-sectional surveys, reported that the ORs of hypertriglyceridemia for diabetes were 1.54 (1.01–2.35) in men and 2.02 (1.49–3.10) in women [4]. A nationwide population-based study of over 5 million adults without known diabetes showed that the hazard ratio for incident diabetes in the low HDL-C and high variability group was 1.40 (1.38–1.42) compared with the high HDL-C and low variability group [24]. The reduction in HDL levels may be caused by a mechanism initiated by elevated plasma TGs against a backdrop of insulin resistance. This mechanism involves the facilitation of the transfer of cholesteryl esters from HDL to TG-rich particles through increased catabolism and the activity of cholesteryl ester transfer protein [26]. This study found that the growth trend of the OR was relatively stable with an increase in TG. However, a strong L-shaped relationship was observed between HDL-C and diabetes, and the reduction in the OR tended to be gradual after the plasma HDL-C level reached 1.44 mmol/L. Significant changes in the slope of the association between HDL-C levels and diabetes may be related to a negative feedback model of pancreatic islets. Evidence suggests that HDL plays a direct role in glycemic control by acting on pancreatic beta cells [27]. Hormonal secretion by the pancreatic islets is highly regulated. Insulin secretion lowers blood glucose levels, and decreasing glucose levels would shut down insulin secretion of pancreatic beta cells, which relieves the local inhibition of glucagon secretion of pancreatic alpha cells [28]. The complementary action of hormones secreted by pancreatic beta and alpha cells allows the maintenance of blood glucose at physiological levels.

Notably, there are significant differences between studies regarding the effects of TC and LDL-C on diabetes [4, 15, 29–31]. In a multicenter retrospective cohort study of 28,476 patients with coronary heart disease, high TC and LDL-C levels were risk factors for diabetes, with ORs of 1.08 (1.06–1.11) and 1.06 (1.03–1.10), respectively [29]. In contrast, increased TC and LDL-C levels were beneficial to reduce the prevalence of diabetes in a cross-sectional cohort study of 3,688 participants aged **≥** 50 years [15]. Another observational study of 9,892 patients with hypertension provided completely different results (a U-shaped relation between LDL-C levels and diabetes prevalence) [30], and RCS performed in this study found similar results that TC and LDL-C showed J-shaped associations with diabetes among older adults ≥ 65 years. Moreover, a new insight was added: the observed J-shaped association between TC and diabetes can be explained when the two different shapes of diabetes prevalence risk for LDL-C and HDL-C are combined. The increased prevalence risk of diabetes within the lower TC range (< 4.04 mmol/L) could be mainly attributed to a combination of the high prevalence risk in older adults with low LDL-C levels and the increase in prevalence risk under low HDL-C conditions may have a synergistic effect on the occurrence of this phenomenon. The increase in diabetes prevalence risk in the higher TC range (≥ 4.04 mmol/L) may be due to the reversal of the relationship between LDL-C and diabetes, and when the promoting effect of high LDL-C on diabetes prevalence exceeds the protective effect of high HDL-C levels, the prevalence risk of diabetes increases with the increase of TC.

The cholesterol paradox regarding serum cholesterol levels and the risk of diabetes has been preliminarily discussed in clinical trials of statins [14]. The patterns observed in this study suggest that HDL-C and LDL-C jointly lead to the cholesterol paradox, and the two opposite effects of low and high LDL-C on diabetes seem to play a crucial role. A post-hoc analysis of an intervention trial demonstrated a positive protective effect of rosuvastatin on coronary artery disease or cerebrovascular disease events, while the risk of diabetes increased with a reduction in LDL-C levels. In Everett BM et al.’s study, the risk of type 2 diabetes among participants with an LDL-C < 30 mg/dl increased by 56% compared with participants with LDL-C ≥ 30 mg/dl [32]. Genetic studies have shown that LDL-C-lowering variants of several genes, including *NPC1L1*, *HMGCR*, and *PCSK9*, are directly associated with an increased risk of diabetes [33]. In addition, overexpression of *NPC1L1* in the liver can suppress gluconeogenesis and lower fasting blood glucose and blood glucose levels, whereas inhibiting NPC1L1 with ezetimibe, a lipid-lowering agent, may promote gluconeogenesis [34]. Furthermore, the free-radical theory of aging and disrupted cholesterol homeostasis were used to account for the inverse association between LDL-C levels and cardiovascular mortality in older people [35]. However, it is unclear whether this hypothesis can explain the negative relationship between LDL-C and diabetes in geriatric populations with low LDL-C levels.

### Strengths and limitations

This study has several strengths. First, the associations between different lipid parameters and diabetes prevalence were fully explored, and the contributions of different cholesterol components to the cholesterol paradox were further elaborated. Second, China’s basic public health services projects and the mature information system can provide a large amount of health-related data. Third, the non-linear relationship between blood lipids and diabetes found in this study, particularly the J-shaped relationship between LDL-C, TC, and diabetes, provides a reference for the management of blood lipid levels in older adults.

However, this study had some limitations. First, participants with diabetes were only differentiated by fasting blood glucose rather than by doctors’ diagnoses, which may have caused some bias. Second, type 1, type 2, and other types of diabetes were not distinguished. Type 2 diabetes accounts for more than 90% of all diabetes cases in China [36]. Third, The data analyzed in this study were cross-sectional, thus preventing any conclusive evidence of a causal relationship between risk factors and diseases. Further prospective studies are required to validate the findings of this study. Fourth, this study only included older Chinese adults and the findings from this population should be cautiously generalized to other populations. Fifth, owing to the limited information contained in the database, no data were collected on the use of lipid-lowering drugs, which may be a confounding factor affecting the results.

## Conclusions

In this large-scale cross-sectional study of older Chinese adults, the relationship between blood lipid parameters and diabetes prevalence was not a simple linear correlation, and low TC and LDL-C levels were risk factors for diabetes prevalence among older Chinese adults. The positive correlation between low LDL-C levels and the risk of diabetes may be related to the cholesterol paradox. Therefore, extremely low LDL-C levels should be avoided in clinical lipid management, particularly in patients without diabetes. These findings may provide a new reference for the development of lipid management guidelines. The makers of blood lipid management strategies need to fully consider the role of the cholesterol paradox to avoid adverse events that may be caused by low blood lipid parameters.

### Electronic supplementary material

Below is the link to the electronic supplementary material.

Supplementary Material 1

Supplementary Material 2

Supplementary Material 3

## Data Availability

The data that support the findings of this study are available from Health Commission of Zhejiang Province, but restrictions apply to the availability of these data, because of the security requirement of the Chinese government. Data were used under license for the current study. Data are however available from the authors upon reasonable request and with permission of Health Commission of Zhejiang Province.

## References

[1] Lin X, Xu Y, Pan X, Xu J, Ding Y, Sun X (2020). Global, regional, and national burden and trend of diabetes in 195 countries and territories: an analysis from 1990 to 2025. Sci Rep.

[2] Zheng Y, Ley SH, Hu FB (2018). Global aetiology and epidemiology of type 2 diabetes mellitus and its complications. Nat Rev Endocrinol.

[3] Oraii A, Shafiee A, Jalali A, Alaeddini F, Saadat S, Masoudkabir F (2022). Prevalence, awareness, treatment, and control of type 2 diabetes mellitus among the adult residents of tehran: Tehran Cohort Study. BMC Endocr Disord.

[4] Cui J, Sun J, Wang W, Xin H, Qiao Q, Baloch Z (2017). The association of triglycerides and total cholesterol concentrations with newly diagnosed diabetes in adults in China. Oncotarget.

[5] Karalis DG. Intensive lowering of low-density lipoprotein cholesterol levels for primary prevention of coronary artery disease. Mayo Clin Proc. 2009;84:345–52. 10.1016/S0025-6196(11)60544-2. 10.1016/S0025-6196(11)60544-2 PMC2665980 19339653

[6] Navarese EP, Robinson JG, Kowalewski M, Kolodziejczak M, Andreotti F, Bliden K (2018). Association between Baseline LDL-C Level and Total and Cardiovascular Mortality after LDL-C lowering: a systematic review and Meta-analysis. JAMA.

[7] Aberra T, Peterson ED, Pagidipati NJ, Mulder H, Wojdyla DM, Philip S (2020). The association between triglycerides and incident cardiovascular disease: what is optimal?. J Clin Lipidol.

[8] Krause MR, Regen SL (2014). The structural role of cholesterol in cell membranes: from condensed bilayers to lipid rafts. Acc Chem Res.

[9] Simons K, Ehehalt R (2002). Cholesterol, lipid rafts, and disease. J Clin Invest.

[10] Hu J, Zhang Z, Shen W-J, Azhar S (2010). Cellular cholesterol delivery, intracellular processing and utilization for biosynthesis of steroid hormones. Nutr Metab (Lond).

[11] Cortes VA, Busso D, Maiz A, Arteaga A, Nervi F, Rigotti A (2014). Physiological and pathological implications of cholesterol. Front Biosci (Landmark Ed).

[12] Corrao G, Ibrahim B, Nicotra F, Soranna D, Merlino L, Catapano AL (2014). Statins and the risk of diabetes: evidence from a large population-based cohort study. Diabetes Care.

[13] Navarese EP, Buffon A, Andreotti F, Kozinski M, Welton N, Fabiszak T (2013). Meta-analysis of impact of different types and doses of statins on new-onset diabetes mellitus. Am J Cardiol.

[14] Sattar N, Preiss D, Murray HM, Welsh P, Buckley BM, de Craen AJM (2010). Statins and risk of incident diabetes: a collaborative meta-analysis of randomised statin trials. Lancet.

[15] Wang T-Y, Chang W-L, Wei C-Y, Liu C-H, Tzeng R-C, Chiu P-Y (2023). Cholesterol Paradox in older people with type 2 diabetes Mellitus regardless of lipid-lowering drug use: a cross-sectional cohort study. Nutrients.

[16] Pan L, Yang Z, Wu Y, Yin R-X, Liao Y, Wang J (2016). The prevalence, awareness, treatment and control of dyslipidemia among adults in China. Atherosclerosis.

[17] Zhang Y-L, Wu B-J, Chen P, Wen H-H (2022). The prevalence, awareness, management and influencing factors of diabetes in middle-aged and elderly in China, evidence from the CHARLS in 2015. Med (Baltim).

[18] Cui J, Sun J, Wang W, Yasmeen N, Ke M, Xin H (2018). Triglycerides and total cholesterol concentrations in association with IFG/IGT in Chinese adults in Qingdao, China. BMC Public Health.

[19] Li X, Krumholz HM, Yip W, Cheng KK, De Maeseneer J, Meng Q (2020). Quality of primary health care in China: challenges and recommendations. Lancet.

[20] Dalong Z, Chinese Diabetes Society (2021). Guideline for the prevention and treatment of type 2 diabetes mellitus in China (2020 edition). Chin J Diabetes Mellitus.

[21] Zhou B-F, Cooperative Meta-Analysis Group of the Working Group on Obesity in China (2002). Predictive values of body mass index and waist circumference for risk factors of certain related diseases in Chinese adults–study on optimal cut-off points of body mass index and waist circumference in Chinese adults. Biomed Environ Sci.

[22] Durrleman S, Simon R (1989). Flexible regression models with cubic splines. Stat Med.

[23] American Diabetes Association (2013). Diagnosis and classification of diabetes mellitus. Diabetes Care.

[24] Lee S-H, Kim H-S, Park Y-M, Kwon H-S, Yoon K-H, Han K (2019). HDL-Cholesterol, its variability, and the risk of diabetes: a Nationwide Population-based study. J Clin Endocrinol Metab.

[25] Ahmed HM, Miller M, Nasir K, McEvoy JW, Herrington D, Blumenthal RS (2016). Primary low level of high-density lipoprotein cholesterol and risks of Coronary Heart Disease, Cardiovascular Disease, and death: results from the multi-ethnic study of atherosclerosis. Am J Epidemiol.

[26] Barter PJ (2011). The causes and consequences of low levels of high density lipoproteins in patients with diabetes. Diabetes Metab J.

[27] Wong NKP, Nicholls SJ, Tan JTM, Bursill CA (2018). The role of high-density lipoproteins in diabetes and its vascular complications. Int J Mol Sci.

[28] Weir GC, Bonner-Weir S (2023). Conflicting views about interactions between pancreatic α-Cells and β-Cells. Diabetes.

[29] Yang T, Liu Y, Li L, Zheng Y, Wang Y, Su J (2022). Correlation between the triglyceride-to-high-density lipoprotein cholesterol ratio and other unconventional lipid parameters with the risk of prediabetes and type 2 diabetes in patients with coronary heart disease: a RCSCD-TCM study in China. Cardiovasc Diabetol.

[30] Liu L, Shen G, Huang J-Y, Yu Y-L, Chen C-L, Huang Y-Q (2019). U-shaped association between low-density lipid cholesterol and diabetes mellitus in patients with hypertension. Lipids Health Dis.

[31] Huang J, Lin H, Wang S, Li M, Wang T, Zhao Z (2023). Association between serum LDL-C concentrations and risk of diabetes: a prospective cohort study. J Diabetes.

[32] Everett BM, Mora S, Glynn RJ, MacFadyen J, Ridker PM (2014). Safety profile of subjects treated to very low low-density lipoprotein cholesterol levels (< 30 mg/dl) with rosuvastatin 20 mg daily (from JUPITER). Am J Cardiol.

[33] Lotta LA, Sharp SJ, Burgess S, Perry JRB, Stewart ID, Willems SM (2016). Association between Low-Density Lipoprotein Cholesterol-Lowering Genetic Variants and risk of type 2 diabetes: a Meta-analysis. JAMA.

[34] Kurano M, Hara M, Satoh H, Tsukamoto K (2015). Hepatic NPC1L1 overexpression ameliorates glucose metabolism in diabetic mice via suppression of gluconeogenesis. Metabolism.

[35] Mc Auley MT, Mooney KM (2017). LDL-C levels in older people: cholesterol homeostasis and the free radical theory of ageing converge. Med Hypotheses.

[36] Weng J, Ji L, Jia W, Lu J, Zhou Z, Zou D (2016). Standards of care for type 2 diabetes in China. Diabetes Metab Res Rev.

