# Supplementary material for: Association between blood lipids and diabetes mellitus in older Chinese adults aged 65 years or older: a cross-sectional analysis of residents’ electronic health records
Source: Lipids Health Dis. 2024 Jun 4;23:167. doi: 10.1186/s12944-024-02160-7 (PMC11149314; doi:10.1186/s12944-024-02160-7)
Supplement: Supplementary file 2 — Supplementary Material 2 [file 12944_2024_2160_MOESM2_ESM.docx]

| 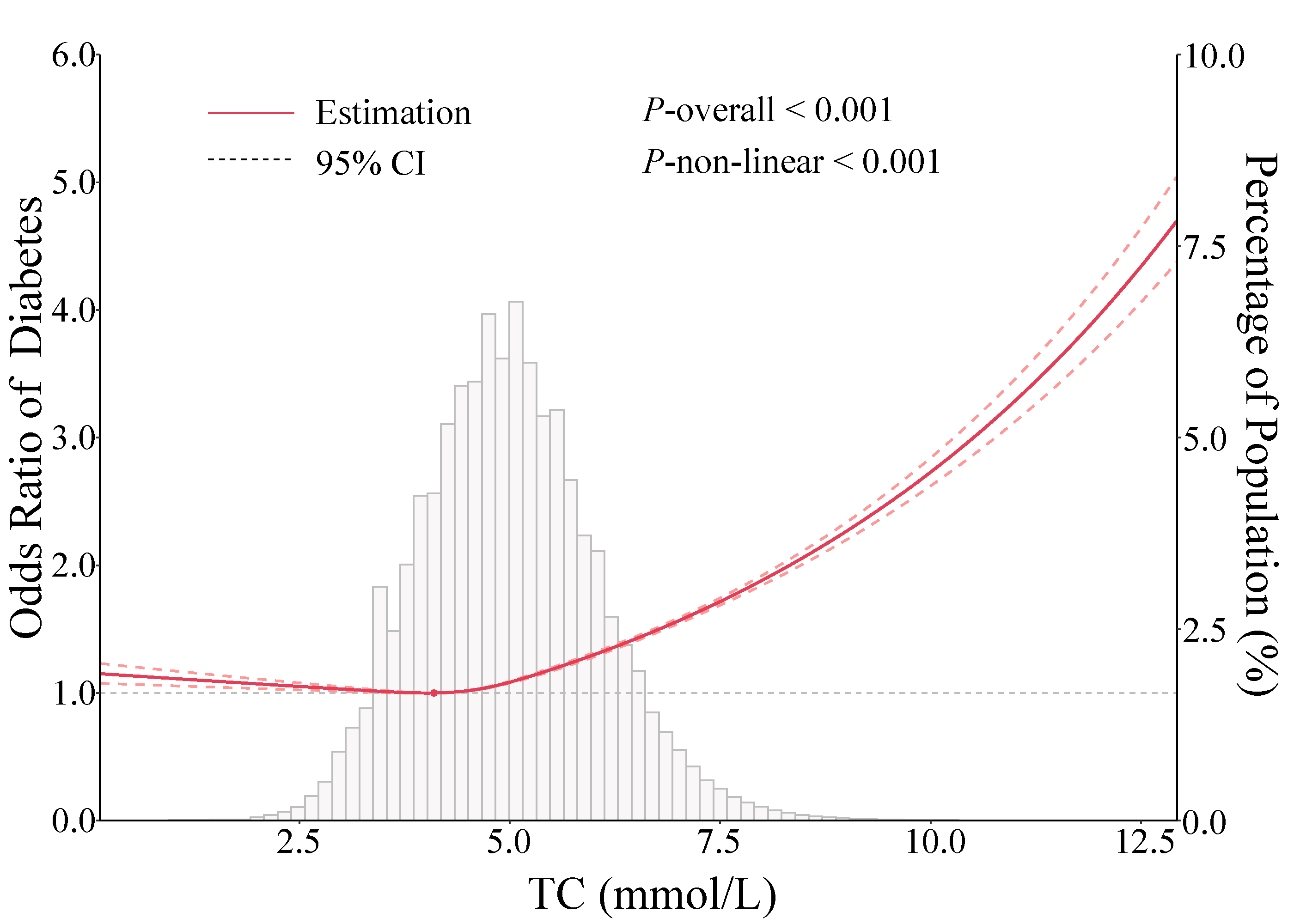  A |
| --- |
| 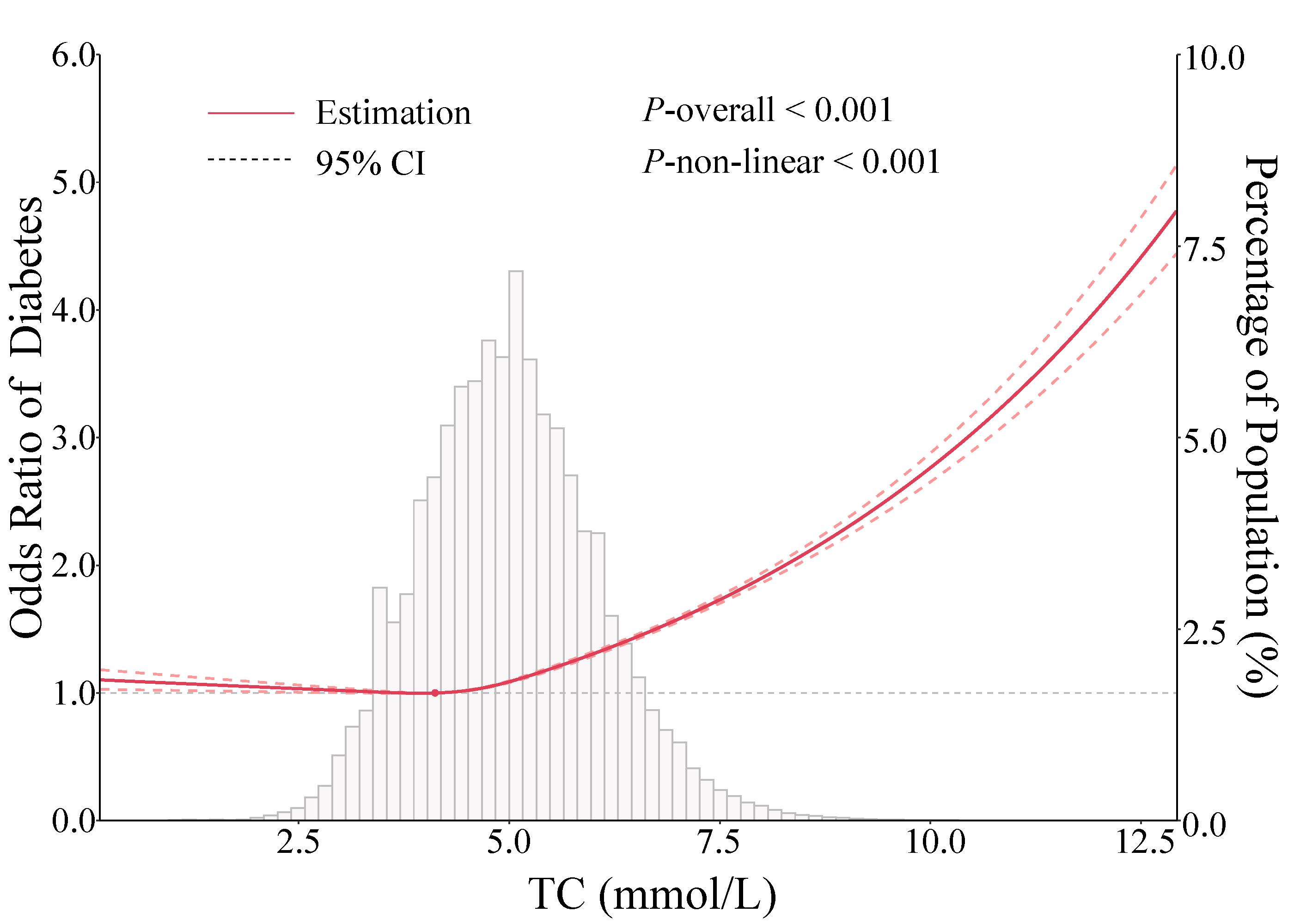  B |
| 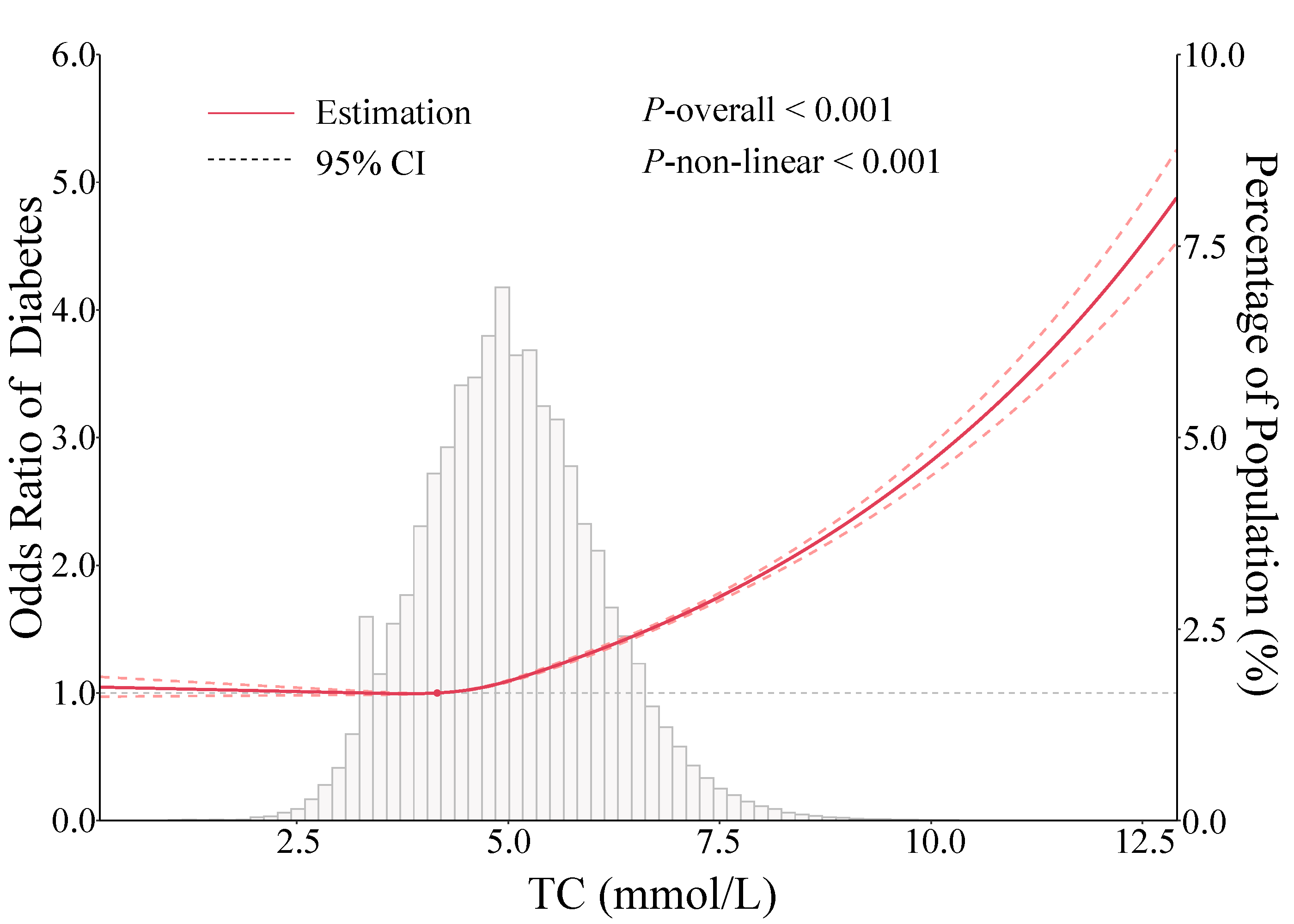  C |

Supplementary Figure A. Odds ratio (95% CI) of diabetes according to TC in older adults aged 65 or older (exclusion of low HDL-C). A: excluded participants with HDL-C below 2.5th centile; B: excluded participants with HDL-C below 5th centile; C: excluded participants with HDL-C below 10th centile. Odds ratios are indicated by red solid lines and border of 95% CIs by red dashed lines (left coordinate axis). Histograms represent the percentage of each group to the total population (right coordinate axis). Reference point is 20th centile of each TC after exclusion of low HDL-C, with knots placed at 5th, 35th, 65th, and 95th centiles. All models were adjusted for age (65-, 70-, 75, 80-), sex (male, female), educational attainment (illiterate and primary, junior and senior, college degree or above, unknown), marital status (single, married, unknown), BMI (normal, low BMI, overweight, obesity), central obesity (no, yes), SBP, DBP, ALT, AST, TBil, Scr, BUN, smoking status (never, regular smoker, former smoker), physical exercise (no, yes) and alcohol consumption status (never, drinker, former drinker).

Supplementary Table A. Odds ratio (95% CI) of diabetes prevalence according to TC, TG, LDL-C and HDL-C stratified by sex

|  | Male |  |  | Female |  |
| --- | --- | --- | --- | --- | --- |
|  | OR (95% CI) | *P*-value |  | OR (95% CI) | *P*-value |
| Normal (n) | 1,420,614 |  |  | 1,645,482 |  |
| Case (n) | 97,394 |  |  | 105,438 |  |
| Prevalence (%) | 6.42 |  |  | 6.02 |  |
| TC |  |  |  |  |  |
| 1 (lowest) | 1 (reference) |  |  | 1 (reference) |  |
| 2 | 1.01 (0.99-1.03) | 0.620 |  | 0.96 (0.94-0.98) | <0.001 |
| 3 | 1.08 (1.06-1.10) | <0.001 |  | 0.99 (0.96-1.01) | 0.194 |
| 4 | 1.17 (1.15-1.20) | <0.001 |  | 1.08 (1.06-1.10) | <0.001 |
| 5 (highest) | 1.45 (1.42-1.48) | <0.001 |  | 1.32 (1.29-1.35) | <0.001 |
| TG |  |  |  |  |  |
| 1 (lowest) | 1 (reference) |  |  | 1 (reference) |  |
| 2 | 1.23 (1.21-1.26) | <0.001 |  | 1.21 (1.17-1.24) | <0.001 |
| 3 | 1.48 (1.45-1.52) | <0.001 |  | 1.43 (1.39-1.47) | <0.001 |
| 4 | 1.78 (1.74-1.82) | <0.001 |  | 1.73 (1.69-1.78) | <0.001 |
| 5 (highest) | 2.61 (2.55-2.67) | <0.001 |  | 2.51 (2.45-2.58) | <0.001 |
| LDL-C |  |  |  |  |  |
| 1 (lowest) | 1 (reference) |  |  | 1 (reference) |  |
| 2 | 0.96 (0.94-0.98) | <0.001 |  | 0.93 (0.91-0.95) | <0.001 |
| 3 | 1.00 (0.98-1.02) | 0.801 |  | 0.93 (0.91-0.95) | <0.001 |
| 4 | 1.07 (1.05-1.09) | <0.001 |  | 1.01 (0.99-1.03) | 0.578 |
| 5 (highest) | 1.25 (1.22-1.27) | <0.001 |  | 1.21 (1.18-1.23) | <0.001 |
| HDL-C |  |  |  |  |  |
| 1(lowest) | 1 (reference) |  |  | 1 (reference) |  |
| 2 | 0.92 (0.90-0.93) | <0.001 |  | 0.94 (0.92-0.96) | <0.001 |
| 3 | 0.83 (0.82-0.85) | <0.001 |  | 0.85 (0.84-0.87) | <0.001 |
| 4 | 0.77 (0.75-0.78) | <0.001 |  | 0.77 (0.75-0.79) | <0.001 |
| 5 (highest) | 0.74 (0.72-0.75) | <0.001 |  | 0.71 (0.70-0.73) | <0.001 |

TC, TG, LDL-C and HDL-C were categorized into five groups based on their respective distributions.

Adjusted for age (65-, 70-, 75, 80-), educational attainment (illiterate and primary, junior and senior, college degree or above, unknown), marital status (single, married, unknown), BMI (normal, low BMI, overweight, obesity), central obesity (no, yes), SBP, DBP, ALT, AST, TBil, Scr, BUN, smoking status (never, regular smoker, former smoker), physical exercise (no, yes) and alcohol consumption status (never, drinker, former drinker).

Supplementary Table B. Odds ratio (95% CI) of diabetes prevalence according to TC, TG, LDL-C and HDL-C stratified by age

|  | 65- |  |  | 70- |  |  | 75- |  |  | 80- |  |
| --- | --- | --- | --- | --- | --- | --- | --- | --- | --- | --- | --- |
|  | OR (95% CI) | *P*-value |  | OR (95% CI) | *P*-value |  | OR (95% CI) | *P*-value |  | OR (95% CI) | *P*-value |
| Normal (n) | 1,165,745 |  |  | 929,543 |  |  | 544,820 |  |  | 425,988 |  |
| Case (n) | 73,932 |  |  | 60,263 |  |  | 36,932 |  |  | 31,705 |  |
| Prevalence (%) | 5.96 |  |  | 6.09 |  |  | 6.35 |  |  | 6.93 |  |
| TC |  |  |  |  |  |  |  |  |  |  |  |
| 1 (lowest) | 1 (reference) |  |  | 1 (reference) |  |  | 1 (reference) |  |  | 1 (reference) |  |
| 2 | 0.96 (0.94-0.99) | 0.005 |  | 1.01 (0.98-1.04) | 0.399 |  | 0.98 (0.94-1.01) | 0.184 |  | 1.03 (1.00-1.07) | 0.072 |
| 3 | 1.03 (1.00-1.05) | 0.057 |  | 1.04 (1.01-1.07) | 0.004 |  | 1.03 (1.00-1.07) | 0.056 |  | 1.09 (1.06-1.13) | <0.001 |
| 4 | 1.14 (1.11-1.17) | <0.001 |  | 1.14 (1.11-1.17) | <0.001 |  | 1.13 (1.09-1.17) | <0.001 |  | 1.13 (1.09-1.17) | <0.001 |
| 5 (highest) | 1.39 (1.36-1.43) | <0.001 |  | 1.42 (1.38-1.45) | <0.001 |  | 1.35 (1.31-1.39) | <0.001 |  | 1.36 (1.31-1.41) | <0.001 |
| TG |  |  |  |  |  |  |  |  |  |  |  |
| 1 (lowest) | 1 (reference) |  |  | 1 (reference) |  |  | 1 (reference) |  |  | 1 (reference) |  |
| 2 | 1.19 (1.16-1.23) | <0.001 |  | 1.24 (1.20-1.29) | <0.001 |  | 1.22 (1.17-1.27) | <0.001 |  | 1.25 (1.20-1.30) | <0.001 |
| 3 | 1.42 (1.37-1.46) | <0.001 |  | 1.46 (1.42-1.51) | <0.001 |  | 1.45 (1.39-1.51) | <0.001 |  | 1.53 (1.47-1.59) | <0.001 |
| 4 | 1.70 (1.65-1.74) | <0.001 |  | 1.74 (1.69-1.80) | <0.001 |  | 1.80 (1.73-1.87) | <0.001 |  | 1.85 (1.78-1.92) | <0.001 |
| 5 (highest) | 2.40 (2.34-2.47) | <0.001 |  | 2.58 (2.50-2.66) | <0.001 |  | 2.55 (2.45-2.65) | <0.001 |  | 2.92 (2.80-3.03) | <0.001 |
| LDL-C |  |  |  |  |  |  |  |  |  |  |  |
| 1 (lowest) | 1 (reference) |  |  | 1 (reference) |  |  | 1 (reference) |  |  | 1 (reference) |  |
| 2 | 0.93 (0.90-0.95) | <0.001 |  | 0.96 (0.93-0.99) | 0.005 |  | 0.96 (0.92-0.99) | 0.010 |  | 0.95 (0.91-0.98) | 0.003 |
| 3 | 0.95 (0.92-0.97) | <0.001 |  | 0.99 (0.96-1.02) | 0.524 |  | 0.97 (0.94-1.01) | 0.141 |  | 0.99 (0.96-1.03) | 0.636 |
| 4 | 1.02 (0.99-1.04) | 0.222 |  | 1.05 (1.03-1.08) | <0.001 |  | 1.05 (1.01-1.08) | 0.007 |  | 1.08 (1.04-1.12) | <0.001 |
| 5 (highest) | 1.21 (1.18-1.24) | <0.001 |  | 1.26 (1.23-1.29) | <0.001 |  | 1.24 (1.20-1.28) | <0.001 |  | 1.25 (1.20-1.29) | <0.001 |
| HDL-C |  |  |  |  |  |  |  |  |  |  |  |
| 1 (lowest) | 1 (reference) |  |  | 1 (reference) |  |  | 1 (reference) |  |  | 1 (reference) |  |
| 2 | 0.93 (0.91-0.95) | <0.001 |  | 0.93 (0.90-0.95) | <0.001 |  | 0.93 (0.90-0.96) | <0.001 |  | 0.93 (0.90-0.97) | <0.001 |
| 3 | 0.86 (0.84-0.88) | <0.001 |  | 0.84 (0.82-0.86) | <0.001 |  | 0.84 (0.81-0.86) | <0.001 |  | 0.83 (0.80-0.86) | <0.001 |
| 4 | 0.78 (0.76-0.80) | <0.001 |  | 0.77 (0.75-0.79) | <0.001 |  | 0.77 (0.74-0.79) | <0.001 |  | 0.76 (0.73-0.79) | <0.001 |
| 5 (highest) | 0.73 (0.71-0.75) | <0.001 |  | 0.73 (0.71-0.75) | <0.001 |  | 0.72 (0.70-0.75) | <0.001 |  | 0.70 (0.68-0.73) | <0.001 |

TC, TG, LDL-C and HDL-C were categorized into five groups based on their respective distributions.

Adjusted for sex (male, female), educational attainment (illiterate and primary, junior and senior, college degree or above, unknown), marital status (single, married, unknown), BMI (normal, low BMI, overweight, obesity), central obesity (no, yes), SBP, DBP, ALT, AST, TBil, Scr, BUN, smoking status (never, regular smoker, former smoker), physical exercise (no, yes) and alcohol consumption status (never, drinker, former drinker).

| 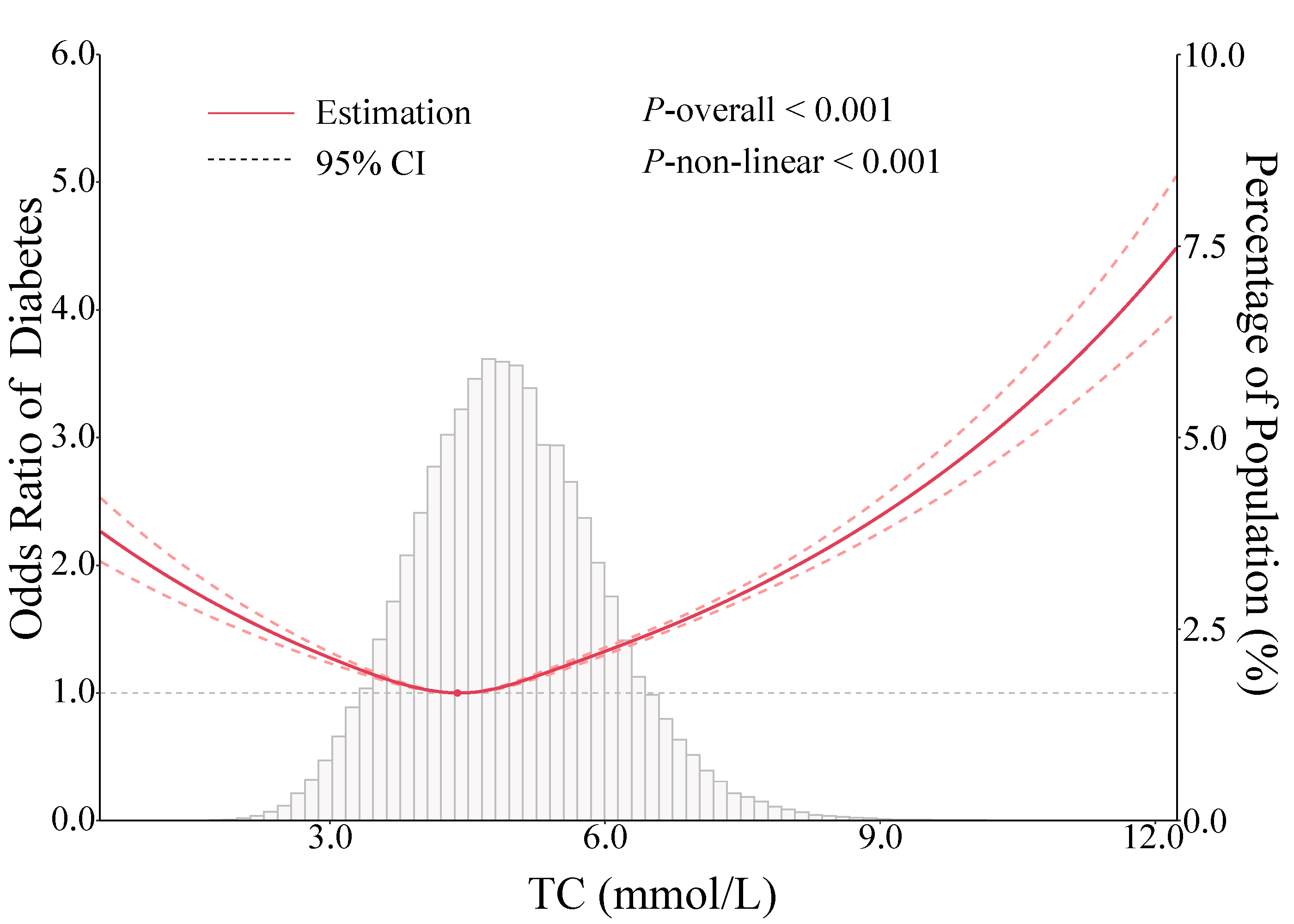  A | 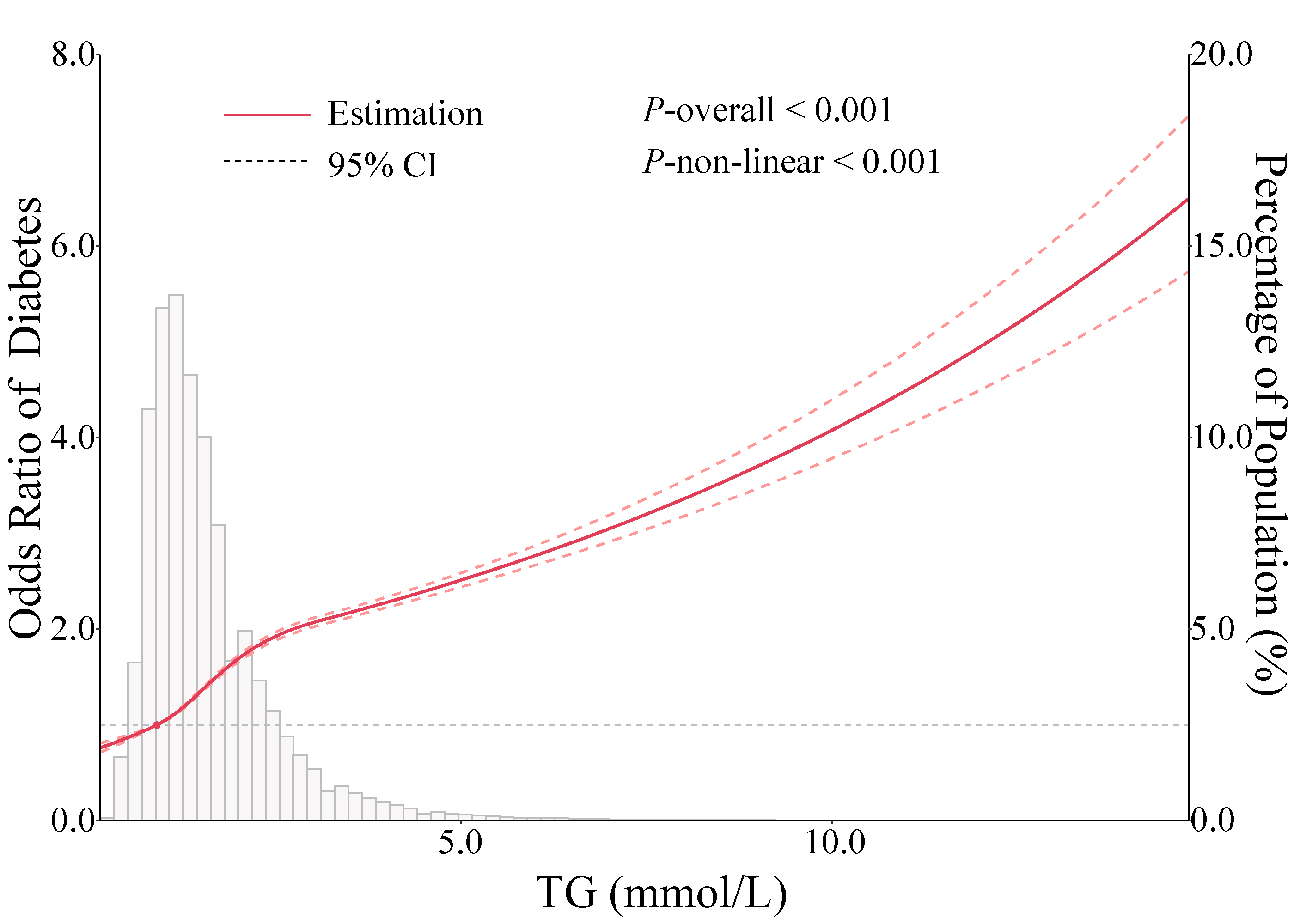  B |
| --- | --- |
| 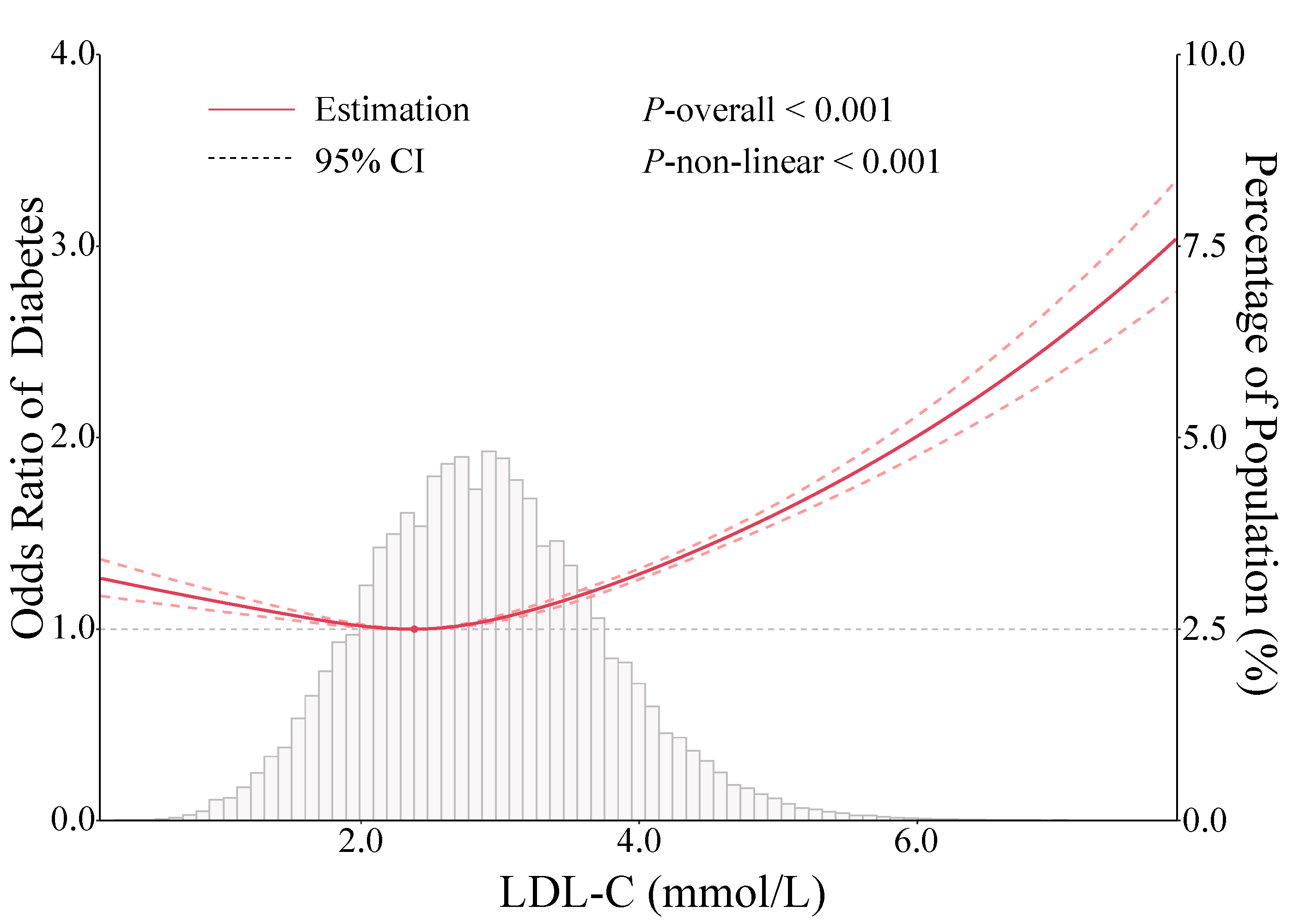  C | 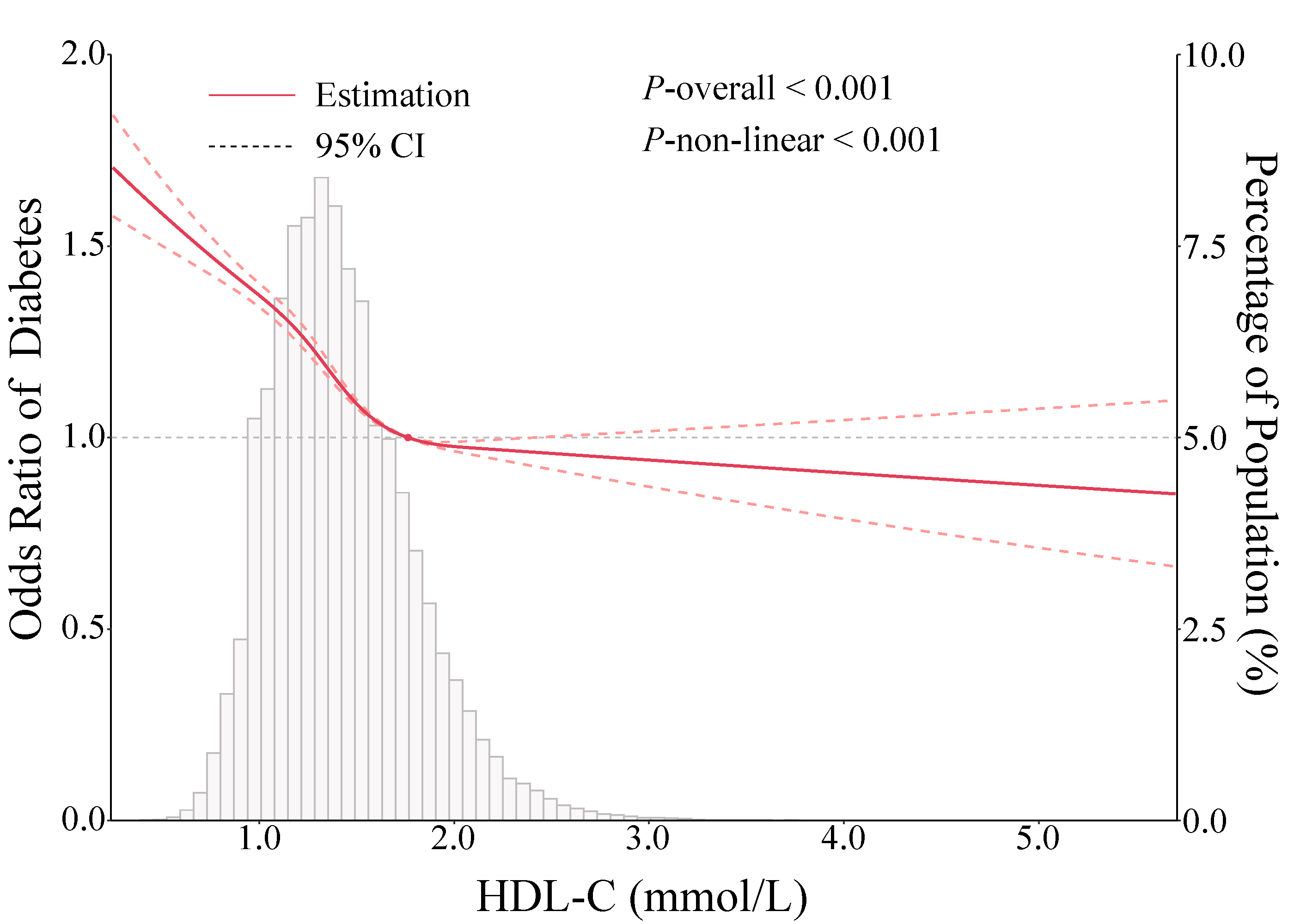  D |

Supplementary Figure B. Association of TC, TG, LDL-C and HDL-C with diabetes (HbA1c ≥ 6.5%) in older adults aged 65 or older. A: TC and diabetes; B: TG and diabetes; C: LDL-C and diabetes; D: HDL-C and diabetes. Odds ratios are indicated by red solid lines and border of 95% CIs by red dashed lines (left coordinate axis). Histograms represent the percentage of each group to the total population (right coordinate axis). Reference point is 20th centile of TG, inflection point of HDL-C and lowest value for each of TC and LDL-C, with knots placed at 5th, 35th, 65th, and 95th centiles of each TC, TG, LDL-C and HDL-C distribution. All models were adjusted for age (65-, 70-, 75, 80-), sex (male, female), educational attainment (illiterate and primary, junior and senior, college degree or above, unknown), marital status (single, married, unknown), BMI (normal, low BMI, overweight, obesity), central obesity (no, yes), SBP, DBP, ALT, AST, TBil, Scr, BUN, smoking status (never, regular smoker, former smoker), physical exercise (no, yes) and alcohol consumption status (never, drinker, former drinker).
